# Ruling out coronary heart disease in primary care patients with chest pain: a clinical prediction score

**DOI:** 10.1186/1741-7015-8-9

**Published:** 2010-01-21

**Authors:** Baris Gencer, Paul Vaucher, Lilli Herzig, François Verdon, Christiane Ruffieux, Stefan Bösner, Bernard Burnand, Thomas Bischoff, Norbert Donner-Banzhoff, Bernard Favrat

**Affiliations:** 1 Department of Ambulatory Care and Community Medicine, University of Lausanne, Switzerland; 2 Institute of General Medicine, University of Lausanne, Switzerland; 3 Institute of Social and Preventive Medicine, University of Lausanne, Switzerland; 4 Department of General Practice/Family Medicine, University of Marburg, Germany

## Abstract

**Background:**

Chest pain raises concern for the possibility of coronary heart disease. Scoring methods have been developed to identify coronary heart disease in emergency settings, but not in primary care.

**Methods:**

Data were collected from a multicenter Swiss clinical cohort study including 672 consecutive patients with chest pain, who had visited one of 59 family practitioners' offices. Using delayed diagnosis we derived a prediction rule to rule out coronary heart disease by means of a logistic regression model. Known cardiovascular risk factors, pain characteristics, and physical signs associated with coronary heart disease were explored to develop a clinical score. Patients diagnosed with angina or acute myocardial infarction within the year following their initial visit comprised the coronary heart disease group.

**Results:**

The coronary heart disease score was derived from eight variables: age, gender, duration of chest pain from 1 to 60 minutes, substernal chest pain location, pain increasing with exertion, absence of tenderness point at palpation, cardiovascular risks factors, and personal history of cardiovascular disease. Area under the receiver operating characteristics curve was of 0.95 with a 95% confidence interval of 0.92; 0.97. From this score, 413 patients were considered as low risk for values of percentile 5 of the coronary heart disease patients. Internal validity was confirmed by bootstrapping. External validation using data from a German cohort (Marburg, n = 774) revealed a receiver operating characteristics curve of 0.75 (95% confidence interval, 0.72; 0.81) with a sensitivity of 85.6% and a specificity of 47.2%.

**Conclusions:**

This score, based only on history and physical examination, is a complementary tool for ruling out coronary heart disease in primary care patients complaining of chest pain.

## Background

Chest pain is a common complaint that occurs in 1 to 2% of primary care patients [1]. Chest pain raises concerns about the occurrence of a serious condition such as coronary heart disease (CHD) [2], which is present in about 12% of primary care patients with chest pain [1,3]. Family practitioners should be equipped to rule out an acute CHD related event rapidly. They are used to estimating the probability of CHD in a patient with chest pain on the basis of pain characteristics, patient's age, gender, history and cardiovascular risk factors [4]. Cardiovascular risk factors and chest pain history are associated with CHD, and have been widely studied [4,5]. However, chest pain characteristics alone are not sufficient to reliably rule out ischemic heart disease [6]. A more complete set of predictors is thus needed for this purpose.

Predictive scores for CHD in emergency settings have been developed [7-9], and are now implemented [10,11]. However, these scores are not necessarily useful in the primary care setting [12]. To our knowledge, no ambulatory CHD score has been developed to support primary care physicians in ruling out CHD in patients with chest pain. Such a score might help physicians, reassure patients [13], and spare time and resources from being spent on further investigations. The objective of this study was to develop an ambulatory CHD predictive score, based only on the patient's history and physical examination in the primary care setting, to rule out CHD without further investigations of patients with chest-pain.

## Methods

### Design overview

We used data from a multicenter clinical cohort of primary care patients with chest pain (TOPIC - Thoracic Pain in Community) [3] to develop a clinical prediction score for CHD, and secondarily the data from a study conducted in Germany (Marburg study) to validate the score [14]. The primary goal of both these studies was to examine the frequency of occurrence, mode of presentation, etiology, clinical characteristics and correlates, management strategies and outcomes in these patients.

### Settings and participants

The initial survey used a research network of family practitioners. Fifty-eight independent offices, urban and rural, and one primary care outpatient clinic from Western Switzerland recruited patients from March to June 2001. Family practitioners consecutively enrolled all patients over 16 years of age who reported any type of chest pain during their visits (n = 672). The presence of chest pain was ascertained according to the usual practice of each family practitioner. Chest pain due to obvious causes such as trauma or known body metastases were also included. Patients with anginal equivalents alone, such as jaw pain, dyspnea on exertion, arm pain, and so on, were therefore excluded. Chest pain was not necessarily the chief complaint on presentation. Participating physicians had an average term of experience in private practice of 12 years (range 1 to 24). They received detailed information on the study and were trained to fill in the forms during a special meeting. Each patient gave informed consent to participate in the study. The study protocol was approved by the official state local Ethical Committee (Prot. 41/2000).

### Data collection

Physicians completed the first part of the Case Report Form (CRF) during the patient's index visit. Additional follow-up information was obtained after three and twelve months (additional encounters). All completed forms were sent to the study coordination center. A set of predefined criteria was used for data entry checks. The data entry clerk reported inconsistencies to the principle investigators, who contacted physicians when needed for case resolution. Missing data were completed by contacting physicians by telephone and obtaining answers from the patient's record. Double data entry was used to identify transcription errors. Data cleaning and validation was performed by a group of physicians experienced in research. When the diagnosis reported by the family practitioner was not consistent throughout the year of follow-up, the final diagnosis for chest pain was discussed and approved by a group of clinicians who were not aware of the aim of this study.

### Predictive factors

We recorded general patients' information as well as type, characteristics and location of chest pain. Chest pain was either already known or a new symptom. An initial plausible etiology, or early diagnosis, was noted. The first part of the CRF included 70 questions on history and clinical signs of chest pain, of which 12 questions concerned factors that were known to be associated with CHD [6], and four concerned factors that were known to be unrelated to CHD (Table 1). These 16 factors of interest were chosen before any analysis as potential predictors of CHD. All variables, except age, were dichotomized, using the usually described cut-off points [6]. Having a cardiovascular risk was defined as having at least one known risk factor (that is, hypertension, hyperlipidemia, diabetes mellitus, smoking, family history of cardiovascular disease (CVD)). Our study then focused on either known pain characteristics related to CHD, or clinical signs excluding CHD. The studied predictors are given in Table 1.

**Table 1:** Distribution of analyzed variables according to diagnostic group and unadjusted Odds Ratio over the derivation cohort.

Variables	Validation cohort	Derivation cohort			
	**(n = 774)**	**All ** **(n = 661)**	**CHD group ** **(*n *= 85)**	**Non-CHD group ** **(*n *= 576)**	**OR_(unadjusted) _** **(95% CI)**
	*%*	*%*	*n *(%)	*n *(%)	
					
***Gender (Male)***	42.0%	47.5%	43 (50.6%)	271 (47.0%)	1.2 (0.7 to 1.8)
***Age-gender categories***					
M < 55 yr or F < 65 yr	48.4%	57.8%	6 (7.1%)	376 (65.3%)	1
M 55 to 64 yr or F 65 to 74 yr	26.0%	14.1%	17 (20.0%)	83 (14.4%)	12.8 (4.9 to 33.5)
M ≥ 65 yr or F ≥ 75 yr	25.6%	27.1%	62 (72.9%)	117 (20.3%)	33.2 (14.0 to 78.7)
***Known CVR***					
None	21.2%	34.5%	2 (2.3%)	226 (39.2%)	1
1-2	53.2%	50.5%	46 (54.1%)	288 (50.0%)	18.1 (4.3 to 15.8)
≥3	25.6%	15.0%	37 (43.5%)	62 (10.8%)	67.4 (15.8 to 287.6)
***Previous history of CVD***	22.0%	18.2%	62 (72.9%)	58 (10.1%)	24.1 (12.5 to 46.4)
***Characteristic of the pain***					
Duration 1 to 60 minutes	42.6%	35.2%	66 (77.6%)	167 (29.0%)	8.5 (4.8 to 15.1)
Increasing on exertion	19.8%	21.3%	37 (43.5%)	104 (18.1%)	3.5 (2.1 to 5.7)
Substernal area pain	57.5%	16.3%	43 (50.6%)	65 (11.3%)	8.0 (4.7 to 13.7)
No tenderness on palpation	57.0%	54.3%	74 (87.1%)	285 (49.5%)	6.9 (3.5 to 13.5)
Sudden excruciating pain	n/a	50.1%	34 (40.0%)	297 (51.6%)	0.63 (0.39 to 1.0)
Oppressive pain	43.4%	36.5%	56 (65.9%)	185 (32.1%)	4.1 (2.5 to 6.7)
Irradiation	14.0%	9.1%	8 (9.4%)	52 (9.0%)	1.0 (0.5 to 2.3)
Not position dependant	n/a	75.8%	77 (90.6%)	424 (73.6%)	3.5 (1.6 to 7.4)
Not increased with breathing	78.2%	76.5%	80 (94.1%)	426 (74.0%)	5.6 (2.2 to 14.4)
*Digestive symptoms*		29.0%	24 (28.2%)	168 (29.2%)	0.96 (0.58 to 1.6)
*Context*					
Known patient	91.3%	91.1%	80 (94.1%)	522 (90.6%)	1.7 (0.64 to 4.3)
New complaint	n/a	48.8%	19/83 (22.9%)	295/561 (52.6%)	0.27 (0.15 to 0.46)
Emergency	n/a	29.1%	19 (22.3%)	173/574 (30.1%)	0.67 (0.39 to 1.1)
Principle complaint	89.3%	52.8%	42/84 (50.0%)	305/573 (53.2%)	0.88 (0.56 to 1.4)
CVR status unknown (no lab)	n/a	4.8%	2 (2.3%)	30 (5.2%)	0.44 (0.1 to 1. 9)

CHD = coronary heart disease; CVD = cardiovascular disease; CVR = cardiovascular risk; OR = odds ratio

### Outcomes and follow-up

In a primary care setting, it is very difficult to have all patients' cardiovascular status assessed using a gold standard. We therefore opted to use a delayed diagnosis over one year to detect patients with CHD. During the initial visit, the suspected diagnosis was noted and then confirmed or modified during follow-up. Detailed information on patients' history and physical examination, level of anxiety expressed by patients and physicians, cardiovascular and thrombo-embolic risk factors, laboratory results obtained in emergencies, co-morbidities, medications, and treatment decisions at the end of the consultation were also collected. CRFs included information on further examinations and laboratory assays, referrals to specialists, admissions to emergency wards, hospitalizations, and health events during the follow-up period. The diagnoses retained after 12 months of follow-up were grouped in six categories: chest wall, CHD, psychogenic, respiratory, digestive, and miscellaneous. CHD included angina pectoris, unstable angina, and myocardial infarction (MI). When the diagnosis of chest pain was inconsistent or uncertain through the follow-up, a group of investigators discussed the case. When the group of investigators was unable to confirm the diagnosis, or if the diagnosis at 12 months was missing, the patient was contacted for further information through his family practitioner. If the patient could not be contacted, the diagnosis at three months was retained. Ten percent of all CRFs were revised by the group of investigators in evaluating the consistency of the final diagnosis.

### External validity

External validity was done using data from a German (Marburg) study. Briefly, 1,199 patients aged 35 and over with chest pain observed in one of 74 family practitioners' offices in Germany were successively included in this study. Chest pain was previously unknown and present for a maximum of one month. Patients were followed-up during six months. Cases were defined as patients having been diagnosed with CHD during the following six months by experts who were blinded to patients' clinical conditions. Variables used from this study were age, sex, cardiovascular risk, history of CVD, presence of retrosternal chest pain, pain triggered by exertion, and pain at palpation. Having a cardiovascular risk was defined as having at least one of the following characteristics: family history of CVD, diabetes, hypertension or treated hypertension, hyperlipidemia or treated hyperlipidemia, smoking or obesity (Body Mass Index ≥30).

### Statistical analysis

From previous studies we expected a prevalence of CHD of 10%. The study was powered to detect factors for which the risk of CHD was increased by two (20% vs. 10%) with β set at 0.2 and α at 0.05. The smallest expected exposure group was the one including patients with previous history of CVD (20%). This required including 127 patients with history of CVD and 510 without, or a total of 637 patients with chest pain. Expecting 10% lost to follow-up or missing data, we rounded this to 700 patients. Bivariate analyses were performed to identify factors associated with CHD in patients with chest pain. Fishers' exact test was used. Variables associated with CHD (*P *< 0.1) were eligible for inclusion in a multivariable logistic model. A variable was retained in the model if it significantly contributed to the model (*P *< 0.05). Goodness of fit was assessed by means of the Hosmer and Lemshow test. A score was then defined on the basis of the coefficients of the logistic regression, and rounded to the nearest unit. Area under the receiver operating characteristic (ROC) curve was an indicator of the discriminatory power of the score. The coefficient of the logistic model with CHD as the dependent variable and the score as the unique independent variable was estimated as a second performance index [15]. Risk categories were defined on the basis of two cut off values of the score: percentile 5 of the CHD group for the *low-risk *category, and percentile 95 of the non-CHD group for the *high-risk *category. Patients with a score between these two cut-off values were considered at *intermediate-risk*. The sensitivity and specificity of the prediction rule for discriminating low- from intermediate- or high-risk cases indicated the classification performance of the score. The prevalence of CHD at given score levels and risk categories was determined, and the mean of the estimated individual probabilities was given. Sensibility analysis was performed to assess the effect of not considering patients with unknown cardiovascular risk status to be at risk. Data were reanalyzed, considering patients with unknown cardiovascular risk status as patients having a risk.

Internal validity was assessed by means of bootstrapping techniques [16]. The whole analysis was replicated on 300 different samples of the same size drawn with replacement from the original sample. Following the same algorithm, new scores and new prediction rules (based on new cut off values) were defined and applied to the original sample and also to the bootstrapping sample. The area under the ROC curve was computed, as well as the calibration index and the sensitivity and specificity of the rule to classify low risk patients. The mean and standard deviation (SD) of the areas under the curves of the 300 scores, applied to the bootstrap samples and to the original sample, mean and standard deviation of the calibration index, applied to the bootstrap sample and to the original sample, and the mean and SD of the sensibilities and specificities of the 300 rules applied to the original sample were obtained. The mean number of times that patients, originally classified as *low-risk*, were reclassified in the low-risk category, and then the mean and SD of the predicted values were computed for each patient. Finally, the mean of the mean and SD of the predicted values were given for each score level and risk category.

For external validation, the area under the ROC curve, sensitivity, specificity, negative likelihood ratio and occurrence of CHD in each risk group were calculated from the data available from the Marburg Study [14]. Only patients with complete data were included in analysis. All calculations were performed with StataCorp. 2008, Statistical Software: Release 10.0., Stata Corporation, College Station, Texas, USA.

## Results

Altogether, 672 patients were included in the study. Twenty-seven patients were lost to follow-up at one year. Their CHD status reported at three months was therefore carried forward and these patients were included in the analysis. Excluded patients were those having missing data on baseline cardiovascular risk status (n = 6) or were younger than 16 (n = 5). In the remaining group of 661 patients, an average of 11.2 (SD, ±7.6) patients were recruited in each of the 59 centers. The total practice-year we followed was of 4.8 years including Thursdays, Saturdays and Sundays when practices are closed. This corresponds to an average of 0.7 patients with chest pain for every GP for every working day. The recruitment period was nevertheless not the same between physicians. Eleven physicians stopped recruiting before they were meant to. If we overview the intervals from which patients were recruited for each physicians, inclusion was constant over time. Physicians apparently either stopped recruiting or continued recruiting per protocol. The mean age of patients included in analysis was 55.4 years (SD ±19), and 314 patients (47.5%) were male. Most patients (602, 91.1%) were already known to their family practitioners, and 314 (47.5%) had never experienced the same type of pain before. One hundred and twenty patients (18.2%) had previous evidence of CHD or CVD (revascularization procedures, heart failure, or peripheral arterial disease) and 433 patients (65.5%) were known to have one or more cardiovascular risk factors, 196 were known to have none. The presence of cardio-vascular risk was unknown to the physician for 32 of the 661 patients. Eighty-five patients (12.9%) were diagnosed with CHD, including 75 cases of angina pectoris (11.1%), six cases of unstable angina (0.9%) and four cases of myocardial infarction (0.6%). None of the 196 patients for which it was known that they had no risk factor, and two of the 32 patients with unknown laboratory status were diagnosed with CHD.

### Building the prediction score

Bivariate analyses identified 11 variables associated with CHD at a significance level of *P = *0.1 (Table 1). The variables were gender, known cardiovascular risk, past history of CVD, substernal pain location, duration of pain, pain during exertion, absence of tenderness, oppressive pain, sudden excruciating pain, pain dependent of the position, pain increased by a deep breath and previously having felt the same pain. Not knowing if patients had dyslipidemia or diabetes (n = 32) was not associated to having CHD (Odds Ratio = 0.44; CI 95% 0.1 to 1. 9).

Age and gender were combined in a single ordinal variable; men <55 and women <65 made the reference group. Men from 55 years to 64 years and women from 65 years to 74 years were coded 1, and men 65 years or over and women 75 years or over were coded 2. Previously having a similar complaint was confounded by other factors (β = 0.59; *P *= 0.131). Including all 661 patients in the regression model, the following variables were not significant at a *P *= 0.05 level: previously having a similar complaint (β = 0.35; *P *= 0.509), not position dependent (β = 0.35; *P *= 0.509), sudden excruciating pain (β = -0.49; *P *= 0.159), oppressive pain (β = 0.65; *P *= 0.070), and not increased by a deep breath (β = 1.3; *P *= 0.059). These variables were excluded, and the coefficient of regression was computed for the seven remaining predictors (Table 2). Goodness-of-fit test of the model showed a good fit between expected and observed frequencies of covariate patterns (*P *= 0.250). The absence of any clustering effect for physicians was confirmed using random-effects logistic regression (ρ = 0.0112; *P *= 0.336). Adjusting for clustering had no effect on CHD score values. Only two cases were not identified at three months and were reported as such during the follow-up period. Results of the logistic regression excluding these two cases remain the same.

**Table 2:** Regression coefficients, contributions to the CHD-score, ORs (adjusted) for the subpopulation with cardiovascular risks (n = 435).

Variables	Regression Coefficient	Score	OR_adjusted _* OR_adj _(95% CI)
**Age-sex categories**			
M < 55 yr or F < 65 yr	0	0	1 (reference)
M 55-64 yr or F 65 to 74 yr	1.99	2	7.3 (2.4 to 22.5)
M ≥ 65 yr or F ≥ 75 yr	2.44	2	11.5 (4.2 to 31.5)
**Known cardiovascular risk**			
None	0	0	1 (reference)
1 to 2	1.76	2	5.8 (1.2 to 29.0)
≥3	1.91	2	6.7 (1.3 to 35.2)
**Known previous history of CVD**	1.89	2	6.7 (3.2 to 13.8)
**Duration of chest pain 1 to 60 minutes**	1.09	1	3.0 (1.4 to 6.2)
**Area of pain described as substernal**	1.65	2	5.2 (2.5 to 10.9)
**Precipitating with exertion**	0.75	1	2.1 (1.0 to 4.3)
**Absence of tenderness**	1.22	1	3.4 (1.5 to 8.0)

* Adjusted for variables shown in this table.

CHD = coronary heart disease; CVD = cardio-vascular disease; OR = odds ratio;

ORadj = adjusted odds ratio

The regression coefficients, OR (adjusted and unadjusted) are given in Table 2, as well as the contribution of each factor to the CHD score. The score ranged from 0 to 11. The model allowed two points each for the following determinates: having a known cardiovascular risk, men over 55 years or women over 65 years, personal history of CVD, and substernal pain. The other determinants, duration, exertion with effort, and tenderness, each received one point.

**Figure 1:**
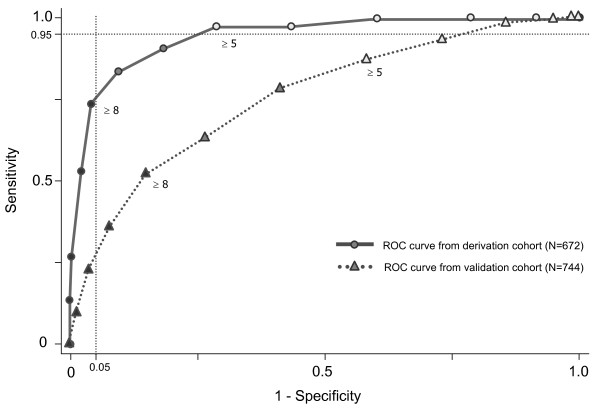
**Receiver operating characteristics curve for the Ambulatory CHD score in both derivation and validation cohorts**.

The area under the curve (AUC) of the receiver operating characteristics (ROC) (Figure 1) was 0.946 (95% CI, 0.924 to 0.968). The model showed a good overall performance (R^2 ^= 0.538). The cut-off point for low-risk (defined as the fifth percentile of the CHD patients) was 5. Thus, the 413 (62.5%) patients with scores <5 were defined as *low-risk*. In the low-risk group, one patient was diagnosed with stable angina and another with myocardial infarction during the following 12 months. The sensitivity of this rule is 97.6% (83/85), the specificity 71.3% (411/576), the negative likelihood ratio of 0.033, and the negative predictive value of 99.5% (411/413).

The cut off point for *high-risk *(defined as the 95^th ^percentile of the non-CHD group) was 7. According to this rule, 87 patients (13.2%) with scores >7 were defined as being at *high-risk*. Finally, 161 patients (24.4%) were classified as intermediate-risk. The prevalence of CHD in the low-risk category was 0.5% (2/413), 12.4% (20/161) in the moderate category and 72.4% (63/87) in the high-risk category (Table 3).

**Table 3:** Prevalence of events for each level of risk.

Events	Low-risk CHD Score = 0-4 *n *= 413			Intermediate-risk CHD Score = 5-7 *n *= 161			High-risk CHD Score = 8-11 *n *= 87		
	
	*n*	%	(CI 95%)	*n*	%	(CI 95%)	*n*	%	(CI 95%)
***Coronary Heart Disease***									
*Observed*	2	0.5%	(0.0 to 1.7)	20	12.4%	(7.8 to 18.5)	63	72.4%	(61.8 to 81.5)
Prevalence from bootstrapping*	0.7 (0.6)			14.6 (10)			69.4 (16)		
Validation cohort (N = 774) †	15/289	5.2%	(3.0 to 8.4)	40/327	12.2%	(8.9 to 16.3)	59/158	37.3%	(29.8 to 45.4)
***Acute myocardial infarction***	1	0.2%	(0 to1.3)	1	0.6%	(0 to 3.4)	2	2.3%	(0.3 to 8.1)
***Unstable angina***	0	0%	(0 to 0.9)	2	1.2%	(0.2 to 4.4)	4	4.6%	(1.3 to 11.4)
***Deaths***									
Overall	4	1.0%	(0.3 to 2.5)	11	6.8%	(3.5 to 11.9)	9	10.3%	(4.8 to 18.7)
CVD	0	0%	(0 to 0.9)	2	1.2%	(0.2 to 4.4)	9	10.3%	(4.8 to 8.7)
CHD	0	0%	(0 to 0.9)	2	1.2%	(0.2 to 4.4)	7	8.0%	(3.3 to 15.9)

Mean and SD of predicted values according to the risk category (bootstrapping).

* Mean prevalence in each risk category (with SD) given by the 300 bootstrap rules (%)

† Data drawn from the German cohort (Marburg study)

CHD = coronary heart disease; CVD = cardio-vascular disease

### Classification of major events and electrocardiograms (ECG)

Table 3 shows the prevalence of major events for each risk class. We observed one patient with acute myocardial infarction classified as low-risk. This patient's cardiovascular risk status was unknown. During the year of follow-up, 24 patients died. Four of them belonged to the *low-risk *group. None of these patients were diagnosed with a CHD. It is worth mentioning that 72 ECGs were taken of low-risk patients, representing 50.3% of all the ECGs done on patients with chest pain. For high-risk patients, less than one third were investigated by ECG.

### Internal validity

The results of the 300 iterations of score development (bootstrapping) gave consistent results with the initial analysis, except for 40 samples. The mean area under the curve for the 300 different prediction rules applied to the original sample was 0.942 (SD 0.004), and the mean calibration index was 0.87 (SD 0.13). When the predictive rules where applied to the bootstrap samples, we had a mean area under the curve of 0.950 (SD 0.011), and a mean calibration index of 0.97 (SD 0.07). The difference between both calibration indexes revealed the *over-optimism *of the model. The dispersion of the calibration index revealed some kind of instability of the model (due to the very small number of CHD in the group without known risk factor). Mean sensitivity and specificity for the rule-out threshold values were 0.956 (SD 0.027) and 0.710 (SD 0.077), respectively.

Nevertheless, the classification appears to be relatively stable because the 392 patients in the low-risk category were classified on average 283.35 times (range 149 to 300) in the low-risk category by the bootstrap rules. The mean prevalence of CHD in the low-risk category was 0.005 (SD 0.005). The distribution of the prevalence of CHD by risk category is given in Table 3 where the score distribution is compared with the actual prevalence. The mean predicted values and mean deviation from original predicted values are given by risk category.

### External validity

**Figure 2:**
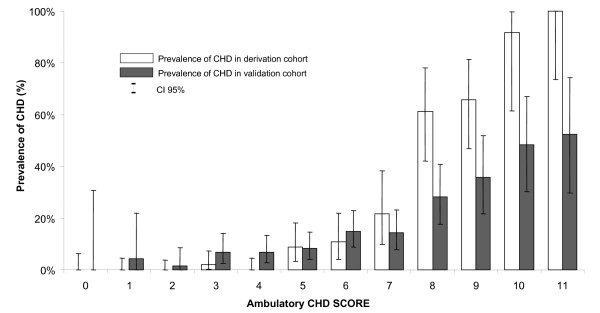
**Observed prevalence of CHD in both derivation and validation cohorts for each ambulatory CHD score value**.

Data from the Marburg study were made available for validation [14]. From the data collected from 1,199 successive patients with chest pain attending 74 general practitioners full data were available for 774 patients that were included in the analysis. Prevalence of CHD was similar (*P *= 0.254) between patients included in analysis (14.7%) and those who were excluded for missing data (12.3%). The area under the curve was of 0.752 (95% CI 0.716; 0.809). Prevalence of CHD in the low risk category (score <5) was of 5.2% (Table 3). The ruling out of CHD using the CHD-Score has a sensitivity of 85.6% and a specificity of 47.2%. Negative likelihood ratio was of 0.305 and negative predictive value of 94.8%. The prevalence of CHD for each score value is given in Figure 2. Among the 15 patients with CHD in the low risk group, one had acute myocardial infarction and one had unstable angina.

## Discussion

We developed a clinical prediction score for ruling out CHD in primary care patients with chest pain. Our CHD score includes eight predictors (age, gender, having at least one CVD risk factor, history of CVD, duration of pain, substernal location of pain, increased pain with exertion, absence of tenderness at palpation), which are all known to be associated with CHD [6], and easily identifiable from history and physical examination. Our score can classify patients in three groups with an increasing prevalence of CHD. Bootstrapping, the recommended tool for this kind of modeling [15], was used to test internal validity. Our ambulatory CHD score is consistent throughout our data, and the predictive power of the model is acceptable. Our ruling-out score classified two thirds of the studied population in the low-risk category with a strong negative predictive value (99.8%). Negative predictive value remained high in the validation cohort (94.8%) with a negative likelihood ratio of 0.31. Our ambulatory CHD score seems adapted for the management of CHDs in primary care. However, this score does not entirely rule out CHD as 5.2% of patients in the low risk group from the validation cohort ended up having CHD within the next six months.

Our data show both similarities and differences from the published determinants used to rule out CHD. The American College of Physicians [4] developed guidelines to manage patients in primary care for whom chronic stable angina was suspected. Our scores are in agreement with their recommendations of using age, gender, cardiovascular risk factors, and pain characteristics to estimate the probability of CHD. Like the Framingham risk score [5], our ambulatory CHD score also includes age, gender, and cardiovascular risks as indicators of CHD events. The Framingham risk score does not include a personal history of CVD or characteristics of pain, which are essential to detecting patients with CHD. These characteristics have been included in scores developed in emergency departments [7,8,17-25]. However, the prevalence and etiology of CHD differ considerably between these two settings. CHD is about four times less frequent in patients with chest pain in primary care [1] than in those admitted in emergency departments [26]. Furthermore, the severity of CHD differs between these settings. Over 10% of patients hospitalized with chest pain [26,27] have a myocardial infarction, whereas we observed a prevalence of only 0.6% in primary care [3]. Factors that may appear to be important or unimportant in samples with relatively few outcome events may be substantially misleading. For example, pain lasting for more than 60 minutes is very atypical for angina but not so unusual for an acute myocardial infarction. Given the very low prevalence of myocardial infarction in this manuscript, factors that may be very important for diagnosing acute myocardial infarction may be substantially undervalued. This could explain why the area under the curve for predicting CHD seems better in our score than for either the TIMI, the PURSUIT or the GRACE scores [28]. This difference illustrates the importance of developing guidelines and scores specifically for the primary care setting instead of a mere use of scores developed for emergency department patients.

The major strength of our study is the development of a clinical tool for family practitioners, using data collected by a research network in primary care [29]. The use of the ambulatory CHD score, based only on the patient's history and physical examination, allows family practitioners to estimate risks of discharging a patient without further examinations.

Our study has some limitations. We would first like to remind physicians that scores derived from logistic regression cannot identify unusual presentations. Consider the example of a young patient who abuses cocaine, and presenting an acute myocardial infarction. This patient would be classified in the low-risk group [30]. Clinical knowledge and experience therefore still remain essential in ruling out CHD in patients with chest pain [31,32]. Secondly, we used delayed diagnosis instead of referring all patients to a specialist. Logistical resources and patients' unavailability make it very difficult to assure objective confirmation in primary care research. Furthermore, in the derivation cohort, assessors were not blinded to the initial state of exposure when defining cases. However, 28% of the patients have been referred to specialists or to hospitals, and the one-year follow up allows us to reasonably confirm the absence of missed CHD. Furthermore, in cases of doubt, the family practitioner was questioned for further information. In the 10% of records reviewed by experts, only one controversial case was detected and resolved after discussion with the family practitioner. We therefore believe that misclassification is negligible. It should be noted that most patients were known to their primary care physicians, a factor that could have facilitated the diagnostic process. Physicians were therefore not blinded to their patient's condition and could have been more likely to report some signs knowing their patient had cardiac chest pain. Furthermore, we cannot exclude that patients with MI or unstable angina which rapidly became entirely asymptomatic remained undetected. The study is also underpowered to detect any difference in myocardial infarction or unstable angina between the risk groups. Finally, external validation showed the score to be much less sensitive than it was meant to be. Both studies however included different patients. In the Marburg study, young patients were not included (≥35 years vs. ≥16 years), nor were those with posterior chest pain or pain lasting more than one month. The Marburg study therefore excluded patients who were more likely to be at low risk compared to those included in the Lausanne cohort. This method nevertheless shows a better diagnosis ability than recognized laboratory markers such as BNP and NT-proBNP [33] in possible heart failure patients.

## Conclusions

In summary, we developed a clinical prediction score to rule out CHD in primary care patients with chest pain. This clinical tool may limit clinical investigation in patients with chest pain in primary care where the presentation and the management are completely different from the emergency departments. However, further analyses of the proposed score should be conducted in various primary care contexts to evaluate its true benefit better and evaluate its implementation.

## Abbreviations

AUC: area under the curve; BMI: body mass index; CHD: coronary heart disease; CI: confidence interval; CRF: case report form; CVD: cardiovascular disease; ECG: electrocardiogram; OR: odds ratio; TOPIC: thoracic pain in community; ROC: rater operating characteristic; SD: standard deviation

## Competing interests

The authors declare that they have no competing interests.

## Authors' contributions

LH, FV, BB, and TB conceived the design, acquired the data and critically revised the manuscript. BG, PV, CR and BF planned, analysed and interpreted the data, and drafted the manuscript. SB and NDB planned, analysed and interpreted data from the validation cohort, and revised the final manuscript.

## Pre-publication history

The pre-publication history for this paper can be accessed here:

http://www.biomedcentral.com/1741-7015/8/9/prepub
